# Case of Delirium Tremens Treated with Large Doses of the Tincture of Digitalis, New Orleans, Charity Hospital, Service of Prof. Flint.

**Published:** 1861-03

**Authors:** L. E. Profilet

**Affiliations:** Resident Student


### *Case of Delirium Tremens, Treated with large doses of the Tincture of Digitalis, New Orleans, Charity Hospital, Service of Prof. Flint.*

[Reported by L. E. Profilet, Resident Student.]

The patient, William C., a laboring man, aged 34, was admitted into the Charity Hospital, December 30th, 1860.

He stated that he had been in the habit of drinking a great deal for some time, but that he had never had delirium tremens. When he entered he had been drinking for five days, but continued at work. He could not tell how many drinks he took a day.

He was intoxicated when admitted. He appeared very feeble, and had much the appearance of a man with congestive chill.

He did not manifest any symptoms of delirium until the second day after his admission, January 1st. He was not violent during the day, but was, evidently, not in his right mind. He was allowed a little brandy. Half a grain of the sulphate of morphia was ordered.

The morphia did not quiet him, and during the night he was very delirious—so much so that it became necessary for some one to watch him, to keep him in bed.

January 2d, he was still delirious. He slept none the night before. He was ordered two drachms of the tincture of digitalis every four hours, until he slept. He took four doses—in all one ounce—before any effect became manifest. Soon after taking the fourth dose, which was given him at 9, P. M., he went to sleep and slept during the whole night.

In the morning he was perfectly rational, and said he felt well. No medicine was ordered.

The next morning, January 4th, he complained of diarrhœa, for which he was ordered one scruple of bismuth, three times daily. The diarrhœa continued one day longer.

He remained two days longer in the Hospital, when he was discharged quite well.—*New Orleans Med. News and Gazette.*

